# Genetic parameters and genomic breeding values for digital dermatitis in Holstein Friesian dairy cattle: host susceptibility, infectivity and the basic reproduction ratio

**DOI:** 10.1186/s12711-019-0505-3

**Published:** 2019-11-20

**Authors:** Floor Biemans, Mart C. M. de Jong, Piter Bijma

**Affiliations:** 1 Quantitative Veterinary Epidemiology, Wageningen University and Research, P.O. Box 338, 6700AH Wageningen, The Netherlands; 2 Animal Breeding and Genomics, Wageningen University and Research, P.O. Box 338, 6700AH Wageningen, The Netherlands

## Abstract

**Background:**

For infectious diseases, the probability that an animal gets infected depends on its own susceptibility, and on the number of infectious herd mates and their infectivity. Together with the duration of the infectious period, susceptibility and infectivity determine the basic reproduction ratio of the disease ($R_{0}$). $R_{0}$ is the average number of secondary cases caused by a typical infectious individual in an otherwise uninfected population. An infectious disease dies out when $R_{0}$ is lower than 1. Thus, breeding strategies that aim at reducing disease prevalence should focus on reducing $R_{0}$, preferably to a value lower than 1. In animal breeding, however, $R_{0}$ has received little attention. Here, we estimate the additive genetic variance in host susceptibility, host infectivity, and $R_{0}$ for the endemic claw disease digital dermatitis (DD) in Holstein Friesian dairy cattle, and estimate genomic breeding values (GEBV) for these traits. We recorded DD disease status of both hind claws of 1513 cows from 12 Dutch dairy farms, every 2 weeks, 11 times. The genotype data consisted of 75,904 single nucleotide polymorphisms (SNPs) for 1401 of the cows. We modelled the probability that a cow got infected between recordings, and compared four generalized linear mixed models. All models included a genetic effect for susceptibility; Models 2 and 4 also included a genetic effect for infectivity, while Models 1 and 2 included a farm*period interaction. We corrected for variation in exposure to infectious herd mates via an offset.

**Results:**

GEBV for $R_{0}$ from the model that included genetic effects for susceptibility only had an accuracy of ~ 0.39 based on cross-validation between farms, which is very high given the limited amount of data and the complexity of the trait. Models with a genetic effect for infectivity showed a larger bias, but also a slightly higher accuracy of GEBV. Additive genetic standard deviation for $R_{0}$ was large, i.e. ~ 1.17, while the mean $R_{0}$ was 2.36.

**Conclusions:**

GEBV for $R_{0}$ showed substantial variation. The mean $R_{0}$ was only about one genetic standard deviation greater than 1. These results suggest that lowering DD prevalence by selective breeding is promising.

## Background

Infectious disease transmission in a host population is a dynamic process. The probability that an animal is infected depends on its own susceptibility, on the number of infected contact animals (“group mates”), and on the infectivity of those group mates. The number and composition of the infectious contact animals in a population varies over time, since some animals get infected while others recover. Whereas state-of-the-art epidemiological models take this variation into account, it should probably also be taken into account to make optimal genetic inference. However, most genetic studies use linear mixed models that ignore the dynamics of the transmission process in the population, such as the variation in exposure of susceptible individuals to infectious contact individuals. Moreover, studies on host genetic variability commonly connect an individual’s disease status to its own pedigree or genotype only, and therefore capture genetic effects related to susceptibility (or resistance) only [1, 2]. However, individuals probably also differ in infectivity, as suggested for example by the observation of superspreaders, which are animals that infect substantially more contact individuals compared to a typical infectious animal [3]. Recently, studies on selection against disease transmission have started that include the infectivity of the herd mates [4–8].

Genetic inference on infectious diseases can probably be improved by using quantitative genetic models that are founded on epidemiological theory. Moreover, such models would give estimates of genetic variation and breeding values for fundamental epidemiological parameters, such as the basic reproduction number $R_{0}$ (see also below). Such knowledge would also facilitate the prediction of response to selection while accounting for the non-linear nature of infectious diseases, including phenomena such as positive feedback and the eradication of a disease when $R_{0}$ falls below 1 [9].

An animals’ infectivity affects the disease status of other animals, rather than its own disease status. Thus, if the observed variation in infectivity has a genetic component, then infectivity is a so-called indirect genetic effect (IGE). IGE can have a considerable effect on the rate and direction of evolution by natural selection, and on response to selective breeding [10–12]. Hence, IGE can and should be used for the genetic improvement of populations whenever they play a role.

Together, susceptibility, infectivity, and the duration of the infectious period determine the basic reproduction ratio ($R_{0}$), which is the average number of secondary cases caused by a typical infectious individual in a fully susceptible population [13]. $R_{0}$ contains information on the ability of an infection to transmit and establish itself in the population [13], and has a threshold value of 1; if $R_{0}$ is lower than 1, an infectious animal will infect, on average, less than one susceptible animal and the disease will die out with certainty. If $R_{0}$ is higher than 1, an epidemic disease can affect a substantial proportion of the population, while an endemic disease may persist in the population. For endemic diseases, the prevalence at the equilibrium state depends on $R_{0}$, and is (ignoring differences in susceptibility between individuals) equal to $1 - \frac{1}{{R_{0} }}$ when $R_{0}$ is higher than 1. Because $R_{0}$ determines the prevalence of a disease in a group of animals (e.g., a herd), breeding strategies that aim at reducing the prevalence should focus on reducing $R_{0}$, ideally to a value lower than 1 [4].

In this study, we focused on the endemic infectious disease digital dermatitis (DD). DD is a claw disorder that affects (mainly) the hind feet of (dairy) cattle [14, 15]. Typically, affected cattle have a round lesion above the interdigital space next to the heel bulbs [16]. These lesions can be painful and prone to bleed, and can develop filiform papillae or be surrounded by hyperkeratotic skin with hairs longer than normal [15]. Severely affected cows show signs of lameness; they bear their weight on the toes of the affected foot, shake the foot as if in pain, and show reluctance to move [15, 17, 18]. The herd prevalence of DD in 383 dairy herds in the Netherlands was estimated at 21.2% in 2003, and within these herds cow level prevalence estimates ranged from 0 to 83.0% [19]. Thus, DD has an impact on the welfare of cows and, furthermore, causes economic losses for the farmer [20, 21].

The prevalence of DD is affected by many factors, such as herd management, lactation stage, flooring system, climate, and breed [19]. Optimizing management strategies are one way to reduce the DD prevalence on a dairy farm [22]. An additional strategy could be to improve claw health through genetic selection [23–25]. As we will argue, this is best done by selecting for lower $R_{0}$.

The objective of our work was to quantify the genetic variation in host susceptibility, host infectivity, and $R_{0}$ for DD in Holstein Friesian dairy cattle, and to investigate our ability to estimate genomic breeding values (GEBV) for these traits. In addition, for model validation, models with and without genetic variation for infectivity will be compared for their ability to predict whether susceptible (infection-free) claws of an animal get infected.

## Methods

### Phenotype data

Phenotypes for DD were collected on 12 dairy cattle farms in the Netherlands, between November 2014 and April 2015. Two observers (author FB being one of them) visited these farms 11 times with a 2-week interval between visits [26]. On each farm, one of the observers rinsed and scored the hind feet using the method of Relun et al. [27], while the other observer recorded cow ID and DD status. Six distinct classes were scored: skin on which lesions were macroscopically absent (M0), displayed a small lesion of 0 to 2 cm (M1), a lesion of more than 2 cm (M2), a lesion covered by a scab (M3), altered skin with dyskeratotis or surface proliferation (M4), and a small lesion in addition to altered skin (M4.1) [28–30]. These classes were divided into susceptible (M0) and infected (M1, M2, M3, M4, and M4.1). Farmers were not informed on the DD status of the cows, but were allowed to identify and treat affected cows using their normal routine. Phenotypes were collected on 1513 cows, of which 1401 were genotyped (see below). On average, a cow was scored 8.7 times. Table 1 shows several characteristics of the farms enrolled in the study.

**Table 1:** Characteristics of the farms enrolled in the study

Farm	Number of cows examined^a^	Number of cows genotyped^a^	Number of observations^b^	Average $\Delta \varvec{t}$ (days)^c^	Number of foot baths^d^	Prevalence (SD)^e^	
						Cow level	Foot level
A	134	116	11	14	7	78.0 (5.4)	69.6 (6.6)
B	105	101	11	14	0	56.3 (7.5)	46.9 (7.9)
C	159	152	11	14	5	49.7 (2.8)	40.2 (1.9)
D	118	116	11	14	7	57.8 (5.0)	49.2 (5.1)
E	102	90	11	13.55	9	62.8 (5.0)	54.6 (5.4)
F	133	112	10	15.56	10	59.2 (10.0)	48.7 (10.4)
G	100	98	11	14	3	65.6 (8.1)	58.2 (7.6)
H	189	180	11	14	7	64.9 (6.2)	56.7 (5.8)
I	104	75	11	14	0	56.4 (5.1)	45.6 (4.9)
J	88	88	11	14	0	65.8 (10.8)	58.1 (10.9)
K	130	116	9	14	13	63.6 (9.6)	52.5 (8.5)
L	151	147	11	13.90	3	70.9 (7.2)	62.0 (7.7)
Total	1513	1401	129	14.07	64	62.6 (7.5)	53.9 (11.0)

^a^Total number of different cows on a farm

^b^Total number farm visits

^c^Average number of days between two scorings ($\Delta t$)

^d^Total number of footbaths given during the study period

^e^Average percentage of animals and feet scored as infected, standard deviation (SD) in brackets

### Genotype data

The cows were genotyped with the Eurogenomics 10 K chip. A first round of quality control was performed before imputation, following the standard procedure of the breeding company CRV. A marker (single nucleotide polymorphism, SNP) was included only when its call rate was higher than 0.85, the deviation of the observed frequency from the expected Hardy Weinberg equilibrium frequency was less than 0.15, and the minor allele frequency was higher than 0.025. Furthermore, inconsistent genotypes between parents and offspring were set to missing. SNPs that passed the quality control were imputed to a set of 76,438 SNPs based on the Illumina BovineSNP50 chip and a custom chip from the breeding company CRV, with a reference population of more than 1000 animals with genotypes on both chips and the combination of Beagle [31] and PHASEBOOK [32] software. A second round of quality control was performed on the imputed data. A SNP was included only when there was no strong deviation from Hardy Weinberg equilibrium (p > 10^−15^), the missing rate was lower than 0.05, and the minor allele frequency was higher than 0.02. In total, 75,904 SNPs passed the quality control and were included in the analysis.

### Models

In this section, we develop a generalized linear mixed model (GLMM) founded on epidemiological principles, to estimate genetic parameters for susceptibility, infectivity and $R_{0}$. To develop the GLMM, we need to find the probability that a susceptible cow gets infected between two observations. We present an epidemiological model, derive the infection probability from this model, and present the resulting GLMM, building on work of Velthuis et al. [5], Lipschutz-Powell et al. [6], Anacleto et al. [7], Anche et al. [4], and Biemans et al. [8].

**Fig. 1:**
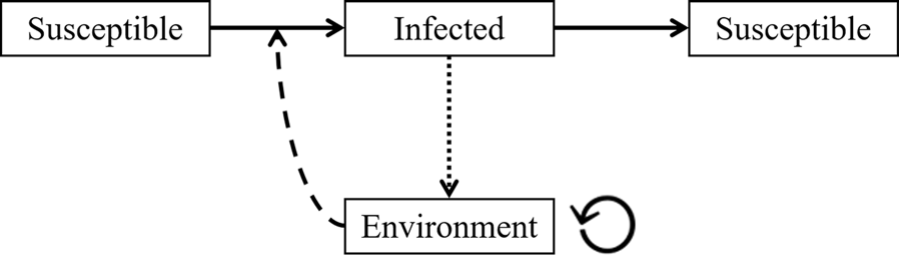
Susceptible-infected-susceptible model with environmental route

DD is an endemic infectious disease with infections reoccurring in the same animals. DD is transmitted via the environment [33], where the environment is defined as any possible pathogen reservoir through which transmission can occur. Thus, we used a stochastic compartmental susceptible-infected-susceptible-model (SIS-model) with an environmental route (E) see e.g. [34] to model disease transmission (Fig. 1). An SIS-model has two categories of individuals: (1) non-infected individuals who can become infected; these are referred to as susceptible; and (2) infected individuals that are also infectious, and who can recover; these are referred to as infected. Hence, the term “susceptible” merely denotes a non-infected individual that can, in principle, become infected. It does not indicate a high degree of susceptibility. In the SIS-model, the infection of a susceptible claw occurs randomly with a probability per observation interval, depending on the parameters of the model, the number of infected claws in the group, and the infection pressure coming from the environment.

In the SIS-model with an environmental route ($E$), the expected rate with which susceptible claws get infected is $\beta S\frac{I + E}{N}$, where $I$ is the number of currently infectious claws, $S$ the number of susceptible claws, and $S + I = N$ the total number of claws in a group (twice the number of cows). Here, it is assumed that infected cows are immediately infectious to their herd mates. $E$ is the infection pressure coming from the environmental reservoir, expressed as the equivalent number of currently infectious claws (i.e., $I$ and $E$ are on the same scale). $\beta$ is the transmission rate parameter that contains information on the contact rate and transmission probability between individuals [35].

Because our interest is in genetic variation in susceptibility and infectivity among cows, we consider the pairwise $\beta$ between a susceptible cow and her infected herd mate. This pairwise $\beta$ depends on the susceptibility of the cow and the infectivity of the herd mate. Thus, the transmission rate parameter $\beta_{ij}$ from an infected claw of herd mate $j$, with infectivity $\varphi_{j}$, to a susceptible claw of cow $i$, with susceptibility $\gamma_{i}$, is:

1 $$ \beta_{ij} = c\gamma_{i} \varphi_{j} . $$

In Eq. 1, the overall contact rate $c$ serves as a scaling parameter, so that mean susceptibility and infectivity are approximately 1, $\overline{\gamma } = \overline{\varphi } = 1$, and mean logarithms are 0, $\overline{\log \left( \gamma \right)} \approx \overline{\log \left( \varphi \right)} \approx 0.$ The latter is relevant for the inclusion of random effects in mixed models, which are commonly assumed to have a mean of 0.

Thus, the expected (i.e., average) rate with which a single susceptible claw of cow $i$ gets infected when exposed to *all* infected claws in the group depends on the susceptibility of cow $i$, on the number of infected claws in the group, and on their average infectivity:

2 $$ Infection\;rate_{i} = c \gamma_{i} \left( {\frac{{\mathop \sum \nolimits_{j} \varphi_{j} }}{{I_{g} }}} \right)\frac{{I_{tot} + E}}{N}, $$

where $c \gamma_{i} \left( {\frac{{\mathop \sum \nolimits_{j} \varphi_{j} }}{{I_{g} }}} \right)$ is the pairwise transmission rate parameter averaged over the genotyped infected herd mates $j$ of susceptible cow $i$, $I_{g}$ is the number of infected claws of herd mates that had genotype records, and $I_{tot}$ is the total number of infected claws at the start of the observation interval. We distinguished between $I_{g}$ and $I_{tot}$ because some of the cows were not genotyped. While we estimated infectivity for the genotyped cows only, the non-genotyped infected cows also contributed to transmission. To account for all infected claws, we assumed that the claws of the non-genotyped cows ($n$ = 112) had the same infectivity as their infected genotyped herd mates ($\mathop \sum \nolimits_{j} \varphi_{j} /I_{g}$).

In the $\frac{{\mathop \sum \nolimits_{j} \varphi_{j} }}{{I_{g} }}$ term (Eq. 2), we should ideally average the infectivity over all claws that contribute to the current infection pressure. This also includes the infection pressure via the environment of the claws that were infected at an earlier time. However, in the statistical software we did not manage to keep track of all the weighted genotypes of claws that were infected at earlier times. Therefore, we only included claws that were infected at the start of the observation interval in the $\frac{{\mathop \sum \nolimits_{j} \varphi_{j} }}{{I_{g} }}$ term. In contrast, the $E$-term included infection pressure of the full *number* of previously infected claws, but these were not weighted according to their infectivity. Thus, our estimates of genetic variation in infectivity use only part of the variation in the infection pressure. This issue is further addressed in the “Discussion” section.

The probability that a susceptible claw becomes infected in an observation interval varies among observation intervals, because it depends on the number of infected herd mates, the infectivity of those herd mates, and on the infection pressure coming from the environment. To estimate this probability, we assumed that the transmission rate (probability per unit of time) is constant within the interval, which is the default assumption in disease transmission models. With this assumption, transmission follows a so-called Poisson process, where the number of transmissions within the interval has a Poisson distribution with a mean equal to the product of the rate and the length of the interval; $\mu = rate \times \Delta t$. A claw becomes a case when it is infected at least once within the interval. Hence, the probability of becoming a case is the complement of the probability of no infection, $P = 1 - e^{ - \mu }$, where $e^{ - \mu }$ is the probability of a zero outcome from a Poisson distribution. Using Eq. 2:

3 $$ P_{i} \left( t \right) = 1 - e^{{ - c\gamma_{i} \left( {\frac{{\mathop \sum \nolimits_{j} \varphi_{j} }}{{I_{g} \left( t \right)}}} \right)\frac{{I_{tot} \left( t \right) + E\left( t \right)}}{N\left( t \right)}\Delta t}} , $$

where, $P_{i} \left( t \right)$ is the probability that a susceptible claw of $i$ is a case (becomes infected) in interval $\Delta t$, given the number of infected herd mates, the infectivity of those herd mates, and on the infection pressure coming from the environment at the start of the interval.

The number of cases for each interval (counting process in discrete time) follows a binomial distribution with a probability that follows from a Poisson process within the interval (counting process in continuous time). Therefore, the complementary log–log is the appropriate link function to connect the explanatory variables to the expected value of the observed variable [36]. Thus, the GLMM follows from applying the complementary log–log transformation to Eq. 3:

4 $$  \begin{aligned} {\text{cloglog}}\left( {P_{i} \left( t \right)} \right) &= \log \left( c \right) + \log \left( {\gamma_{i} } \right) + { \log }\left( {\frac{{\mathop \sum \nolimits_{j} \varphi_{j} }}{{I_{g} \left( t \right)}}} \right) \\&\quad+ \log \left( {\frac{{I_{tot} \left( t \right) + E\left( t \right)}}{N\left( t \right)}\Delta t} \right), \end{aligned} $$

where $\log \left( c \right)$ is an intercept, $\log \left( {\gamma_{i} } \right)$ is the logarithm of susceptibility of the focal individual, ${ \log }\left( {\frac{{\mathop \sum \nolimits_{j} \varphi_{j} }}{{I_{g} \left( t \right)}}} \right)$ the log of the mean infectivity of infected (genotyped) herd mates, and ${ \log }\left( {\frac{{I_{tot} \left( t \right) + E\left( t \right)}}{N\left( t \right)}\Delta t} \right)$ is an offset, i.e., a known “explanatory variable”. The offset accounts for the infectious pressure coming from the infected cows (both genotyped and non-genotyped) at time $t$: ($I_{tot} \left( t \right)/N\left( t \right))$ and from the environment at $t$: ($E\left( t \right)/N\left( t \right)$), and for the length of the interval ($\Delta t$). Note that the dependent variable for Eq. 4 is the number of cases for a cow with at least one susceptible claw. For cows with one susceptible claw, the binomial total equals 1 and the number of cases takes values $C$ = 0 or 1. For cows with two susceptible claws, the binomial total equals 2 and the number of cases takes values $C$ = 0, 1 or 2.

Equation 4 is linear in the logarithm of susceptibility, but not in the logarithm of infectivity. To allow the fitting of a linear model, we linearized the model term for infectivity following [37] in the models in which the infectivity of the group mates was included (Models 2 and 4, see below):

5 $$ \begin{aligned} {\text{cloglog}}\left( {P_{i} \left( t \right)} \right) &\approx \log \left( c \right) + \log \left( {\gamma_{i} } \right) + \frac{1}{{I_{g} \left( t \right)}}\mathop \sum \limits_{j} \log \left( {\varphi_{j} } \right)\\&\quad + \log \left( {\frac{{I_{tot} \left( t \right) + E\left( t \right)}}{N\left( t \right)}\Delta t} \right). \end{aligned} $$

Equation 5 follows from approximating the $\left( {\frac{{\mathop \sum \nolimits_{j} \varphi_{j} }}{{I_{g} \left( t \right)}}} \right)$, which is an arithmetic mean, by the corresponding geometric mean (see [37] for details). The errors caused by this approximation are less than 5% for infectivity effects up to a factor of 3 (i.e., $\varphi_{j}$ between 0.33 and 3.0). However, this error estimation is based on a model with SNP effects rather than polygenic breeding values. The size of the error is not known for a trait determined by many genes each with a small effect. The method will anyway take the real differences in exposure for different observation periods on the different farms into account. We acknowledge that this is a relevant issue that requires further investigation.

We calculated the infectious pressure coming from the environment ($E\left( t \right)$) as described in detail in Biemans et al. [26]. In short, claws that were infected at an earlier time could still contribute partly to the environmental reservoir at the moment of observation. Their contribution was assumed to decrease each interval $\Delta t$ by a factor $\lambda$, which may be interpreted as a survival rate of the pathogen. The estimate for this survival rate is 0.9 [26]. Thus, the number of pathogens in the environment coming from a claw that was infected at $t$, is a fraction 0.9 at $t + 1$, a fraction 0.9^2^ = 0.81 at $t + 2$, a fraction 0.9^3^ = 0.729 at $t + 3$, etc. Therefore, the values for $E\left( t \right)$ were calculated as:

6 $$ E\left( t \right) = 0.9\left[ {I_{tot} \left( {t - 1} \right) + E\left( {t - 1} \right)} \right], $$

where $I_{tot} \left( {t - 1} \right)$ is the total number of infected claws at $t - 1$, and $E\left( {t - 1} \right)$ is the infectious pressure coming from the environmental reservoir at $t - 1$.

Because we did not observe the number of infected claws in the period before the first observation ($I_{tot} \left( {t   0} \right)$), we estimated this number with a linear model. For each farm, we fitted the model to the number of infected claws over the observation period. The intercept of the model, $I_{tot} \left( {t = 0} \right)$, was used as the average number of infected claws on that particular farm in the periods before observations started ($I_{tot} \left( {t   0} \right)$). Thereafter, the value for the environmental reservoir at the first observation was estimated as, $E\left( {t = 0} \right) = \frac{0.9}{1 - 0.9}I_{tot} \left( {t \le 0} \right)$, and was used in Eq. 6.

### Implementation of the model

With the GLMM, we modelled the expectation of the number of cases over the number of susceptible feet (claws) of cow $$ i $$ within the interval $$ \Delta t $$, $$ P_{i} \left( t \right) = E\left( {{\raise0.7ex\hbox{${C_{i} \left( t \right)}$} \!\mathord{\left/ {\vphantom {{C_{i} \left( t \right)} {F_{i} \left( t \right)}}}\right.\kern-0pt} \!\lower0.7ex\hbox{${F_{i} \left( t \right)}$}}} \right) $$. Only the hind feet of the cows were scored, so a susceptible cow could have one or two susceptible feet ($$ F $$) at the start of an interval, which were zero, one, or two cases by the end of the interval. Thus, the number of cases $$ C $$ (0, 1 or 2) for each susceptible cow followed a binomial distribution with binomial total $$ F $$ (1 or 2).

We tested four models (Table 2). Model 1 included a genetic effect for susceptibility only:

$$ \begin{aligned} & {\text{cloglog}}(P_{iklm\tau } \left( t \right)) = c_{0} + Farm_{k} + Period_{\tau } \\ & \quad+ Parity_{l} + c_{1} MIM_{m} + Farm_{k} . Period_{\tau } \\ &\quad + Animal_{i} + { \log }\left( {\gamma_{i} } \right) + { \log }\left( {\frac{{I_{tot} \left( t \right) + E\left( t \right)}}{N\left( t \right)}\Delta t} \right),\quad \left( {\text{Model 1}} \right) \\ \end{aligned} $$

where $c_{0}$ is the intercept. The fixed effects were farm ($Farm_{k}$ with $k$ = A to L), period ($Period_{\tau }$ with $\tau$ = 1 to 10), parity ($Parity_{l}$ with $l$ = 1, 2, or > 2), and months in milk ($MIM_{m}$, a continuous covariate with $m$ = 1 to 12). Random effects included an interaction between farm and period ($Farm_{k} . Period_{\tau }$ with $k$ = A to L and $\tau$ = 1 to 10), a non-genetic permanent animal effect for the susceptible animal $i$ ($Animal_{i}$) to account for repeated observations on the same animal in different periods, and an additive genetic effect for susceptibility of animal $i$ (${ \log }\left( \gamma \right)\sim {\text{N}}\left( {0, {\mathbf{G}}\sigma_{\text{a}}^{2} } \right)$, where ${\mathbf{G}}$ is the genomic relationships matrix among animals).

**Table 2:** Overview of the fixed and random effects included in the four models

Model	Random effects		
1	Genetic susceptibility focal individual	–	Farm*period
2	Genetic susceptibility focal individual	Genetic infectivity herd mates	Farm*period
3	Genetic susceptibility focal individual	–	–
4	Genetic susceptibility focal individual	Genetic infectivity herd mates	–

All models contained fixed effects for farm, period, parity, and months in milk; and a non-genetic random animal effect for the susceptible animal

Model 2 included genetic effects for both susceptibility and infectivity:

$$ \begin{aligned}  & {\text{cloglog}}(P_{ijklm\tau } \left( t \right)) = c_{0} + Farm_{k} + Period_{\tau } \\ &\quad + Parity_{l} + c_{1} MIM_{m} + Farm_{k} . Period_{\tau } \\ &\quad + Animal_{i} + { \log }(\gamma_{i} ) + \frac{1}{{I_{g} \left( t \right)}}\mathop \sum \limits_{j} \log \left( {\varphi_{j} } \right) \\&\quad+ { \log }\left( {\frac{{I_{tot} \left( t \right) + E\left( t \right)}}{N\left( t \right)}\Delta t} \right),\quad \left( {\text{Model 2}} \right) \\ \end{aligned} $$

where $\mathop \sum \nolimits_{j} { \log }(\varphi_{j} )$ are the random genetic effects for infectivity of the infected group mates $j$ of animal $i$, with ($\log \left( \varphi \right)\sim {\text{N}}\left( {0,{\mathbf{G}}\sigma_{\text{a}}^{2} } \right)$).

We expected that the interaction between farm and period could be partly confounded with the genetic effect for infectivity, because previous IGE studies showed that omitting group-effects may substantially inflate estimated genetic parameters for infectivity [38]. To investigate this issue, we dropped the farm by period interaction from Models 1 and 2, resulting in Models 3 and 4, respectively.

### Data analyses

The ${\mathbf{G}}$-matrix was computed using method 1 of VanRaden [39], with the calc_grm software [40]. We fitted the four models with ASReml v4.1.0 [41]. Model fit was assessed with Akaike information criterion (AIC). The susceptibility and infectivity estimates from ASReml were on the log scale because of the complementary log–log link function (Eq. 5). These random effects are zero on average. By taking the exponent of the estimates, we obtained the genomic estimated breeding values (GEBV) for susceptibility and infectivity, relative to a typical (average) individual that has a GEBV of 1 (thus $\overline{\log \left( \gamma \right)} = \overline{\log \left( \varphi \right)} = 0$, therefore $\overline{\gamma } \approx \overline{\varphi } \approx 1$).

### Cross-validation

We validated the GEBV to investigate their bias and accuracy with a 12-fold cross-validation on all four models. In each analysis, all the cases ($C$) of one of the 12 farms were censored from the dataset. For each susceptible claw of cow $i$ at interval $t$ at the censored farm, we predicted the dependent variable of the GLMM, $C_{i} \left( t \right)/F_{i} \left( t \right)$, based on the information of the other 11 farms. In the following, we refer to this prediction as the predicted probability $\widehat{{P_{i} }}\left( t \right)$.

However, as the fixed effects were nonlinear on the normal scale because they were estimated with a complementary log–log link function, correction of the observed records for fixed effects was not straightforward. To solve this issue, we translated both the predicted probabilities and the observed records to a standard (i.e., average) farm. Subsequently, we validated the models using a weighted correlation and a regression of observed records on predicted probabilities (see “Appendix” for detailed methods). To calculate the correlation and regression coefficients, we used observations and predictions averaged over the number of times an animal was susceptible at the start of an interval, and this number was used as the weight.

### Genetic variance and breeding values for the basic reproduction ratio

For the best fitting model (lowest bias, highest accuracy), we calculated the genetic variance and GEBV ($\widehat{A}_{{R_{0} ,i}}$) for the basic reproduction ratio.

The additive genetic variance of $R_{0}$ was calculated as [4]:

$$ \begin{aligned} \sigma_{{A_{{R_{0} }} }}^{2} &= \left( {\overline{\gamma }^{2} \sigma_{\varphi }^{2} + \overline{\varphi }^{2} \sigma_{\gamma }^{2} + \sigma_{\gamma }^{2} \sigma_{\varphi }^{2} } \right)\left( {\frac{c}{\alpha }} \right)^{2} \\ &\approx \left( {\sigma_{\gamma }^{2} + \sigma_{\varphi }^{2} + \sigma_{\gamma }^{2} \sigma_{\varphi }^{2} } \right)\left( {\frac{c}{\alpha }} \right)^{2} , \end{aligned}$$

where the approximation follows from $\bar{\gamma } \approx \bar{\varphi } \approx 1.$

The GEBV for the basic reproduction ratio is the product of the GEBV for susceptibility ($\widehat{\gamma }_{i}$), the GEBV for infectivity ($\hat{\varphi }_{i} )$, the contact rate ($c$), and the average duration of the infectious period ($1/\alpha$) [4]:

7 $$ \hat{A}_{{R_{0} ,i}} = \frac{{\hat{\gamma }_{i} \hat{\varphi }_{i} c}}{\alpha } = \frac{{\hat{\beta }_{i} }}{\alpha }. $$

With a previously estimated $R_{0}$ for DD of 2.36 on these farms [26] and the average product of the estimated relative susceptibility and relative infectivity, the value for $c/\alpha$ was calculated as $c/\alpha = \frac{2.36}{{\overline{{\hat{\gamma }_{i} \hat{\varphi }_{i} }} }}$. The breeding value for $R_{0}$ in Eq. 7 is on the absolute scale, and thus has an average equal to $R_{0}$, rather than zero. This is convenient, because absolute breeding values for $R_{0}$ can be interpreted relative to a value of 1, which is the threshold for eradication of a disease. Note that, in Models 1 and 3, variation in infectivity is not estimated. Hence, for these models we used $\hat{\varphi }_{i} = 1$ for all individuals.

GEBV are prone to overestimation, as illustrated by regression coefficients of validation phenotypes on predicted GEBV, which are often less than 1 e.g. [42]. To avoid overestimation of the variation in breeding values for $R_{0}$, we shrunk the estimates for the transmission rate parameter

$$ \hat{\beta }_{i,corrected} = b\left( {\beta_{i} - \bar{\beta }} \right) + \bar{\beta }, $$

where $b$ is the regression coefficient of the observed $C/F$ on the predicted probability, averaged over cross-validation sets. The corrected individual breeding values for the basic reproduction ratio were:

$$ \hat{A}_{{R_{0} ,i,corrected}} = \frac{{\hat{\beta }_{i,corrected} }}{\alpha }. $$

After this correction, the regression coefficient of the observed $C/F$ on the predicted probability in cross-validation was 1, implying that the GEBV have the correct variance. Moreover, when the regression coefficient of phenotypes on predicted breeding values equals 1, and observations and predictions follow an approximate normal distribution, then the accuracy of EBV equals the ratio of the standard deviations, $cor\left( {\hat{A}_{{R_{0} }} ,A_{{R_{0} }} } \right) = \sigma_{{\hat{A}_{{R_{0} }} }} /\sigma_{{A_{{R_{0} }} }}$ [43]. We used this result to obtain the approximate accuracy of the GEBV for $R_{0}$.

## Results

### Fixed effects

The farm effect was significant (P < 0.05) in Models 1 and 3, but not in Models 2 and 4. For all models, there was a significant effect for period, parity, and months in milk. The probability of becoming infected during an interval increased during the first six periods and stabilized thereafter. The transmission rate parameter increased with increasing parity; it was 21% higher for parity 2 compared to parity 1, and 69% higher for parities greater than 2 compared to parity 1. For months in milk, the transmission rate parameter decreased by 4% with every month in milk. A table of fixed effect estimates and standard errors on the log scale from Model 1 is presented in Additional file 1.

### Estimated variance components

Table 3 shows the estimated variance components and their standard errors (SE) on the log scale. The estimated variance components are similar on the normal scale, because $var\left( {\ln \left( x \right)} \right) \approx \left( {\left[ { \frac{d\ln \left( x \right)}{d x}} \right]_{x = 1} } \right)^{2} var\left( x \right) = \left( {\left[ { \frac{1}{ x}} \right]_{x = 1} } \right)^{2} var\left( x \right) = var\left( x \right)$ around $\ln \left( x \right) = 0$ ($x$ = 1) [44]. For Models 1, 2, and 4, the estimated genetic variance for susceptibility was about 0.55, and was strongly significant. These models had a genetic effect for infectivity and/or the interaction term between farm and period. For Model 3, the estimated genetic variance for susceptibility was smaller (0.49); this model had neither a genetic effect for infectivity, nor a term for the interaction between farm and period. Similarly, the variance of the non-genetic random animal effect was about 0.95 for Models 1, 2, and 4; and was smaller, ~ 0.92, for Model 3. The estimated variance for infectivity was large, and not significant for Model 2. The interpretation of the magnitude of these variance components is discussed below, in the section on $R_{0}$.

**Table 3:** Estimated variance components and their standard errors (SE) for the genetic effect of susceptibility and infectivity, the interaction between farm and period, and the animal effect for the four models

Model	Estimated variance (SE) of the random terms			
	Susceptibility	Infectivity	Farm*period	Animal
1	0.555 (0.142)	–	0.262 (0.050)	0.949 (0.130)
2	0.558 (0.142)	25.43 (16.41)	0.144 (0.067)	0.952 (0.130)
3	0.490 (0.131)	–	–	0.922 (0.123)
4	0.556 (0.142)	68.27 (14.13)	–	0.952 (0.130)

**Fig. 2:**
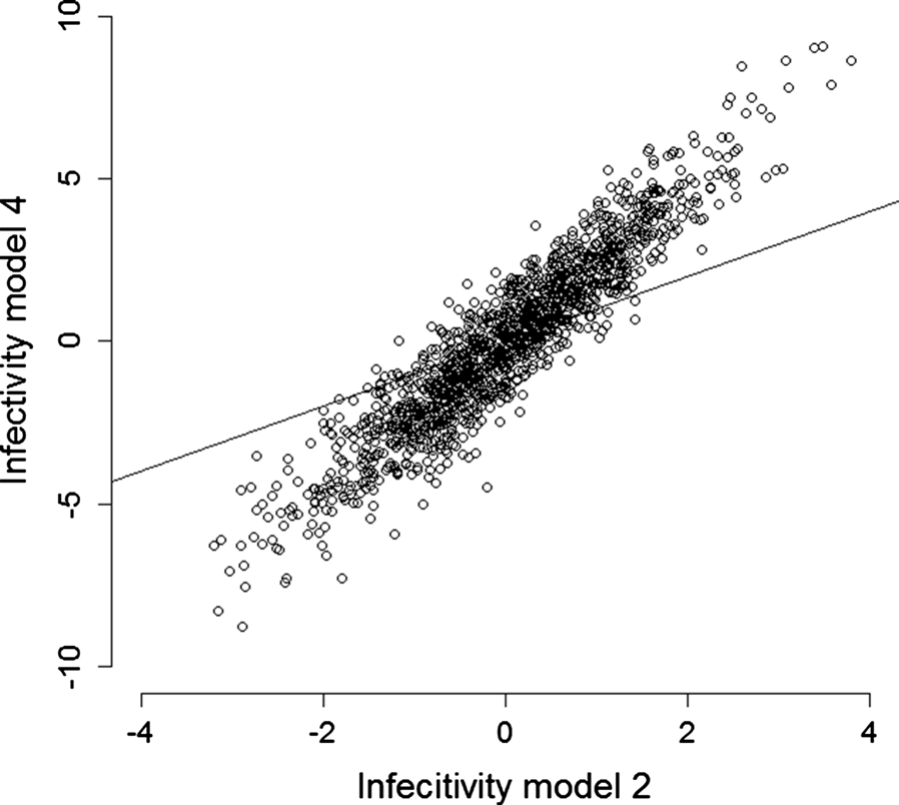
Estimated infectivity effect from Model 2 versus Model 4. Each point represents one cow. The line shows y = x

Figure 2 shows the infectivity estimates from Model 4 plotted against the infectivity estimates from Model 2. The estimates varied from − 3.20 to 3.80 for Model 2 and from − 8.76 to 9.04 for Model 4. Thus, the GEBV for infectivity from Model 2 showed less variation compared to the GEBV from Model 4; part of the variation that is attributed to the interaction between farm and period in Model 2, is attributed to the genetic infectivity effect in Model 4. This suggests that the GEBV for infectivity from Model 4 include both a genetic and a non-genetic component, and may therefore be inflated.

### Cross-validation

**Fig. 3:**
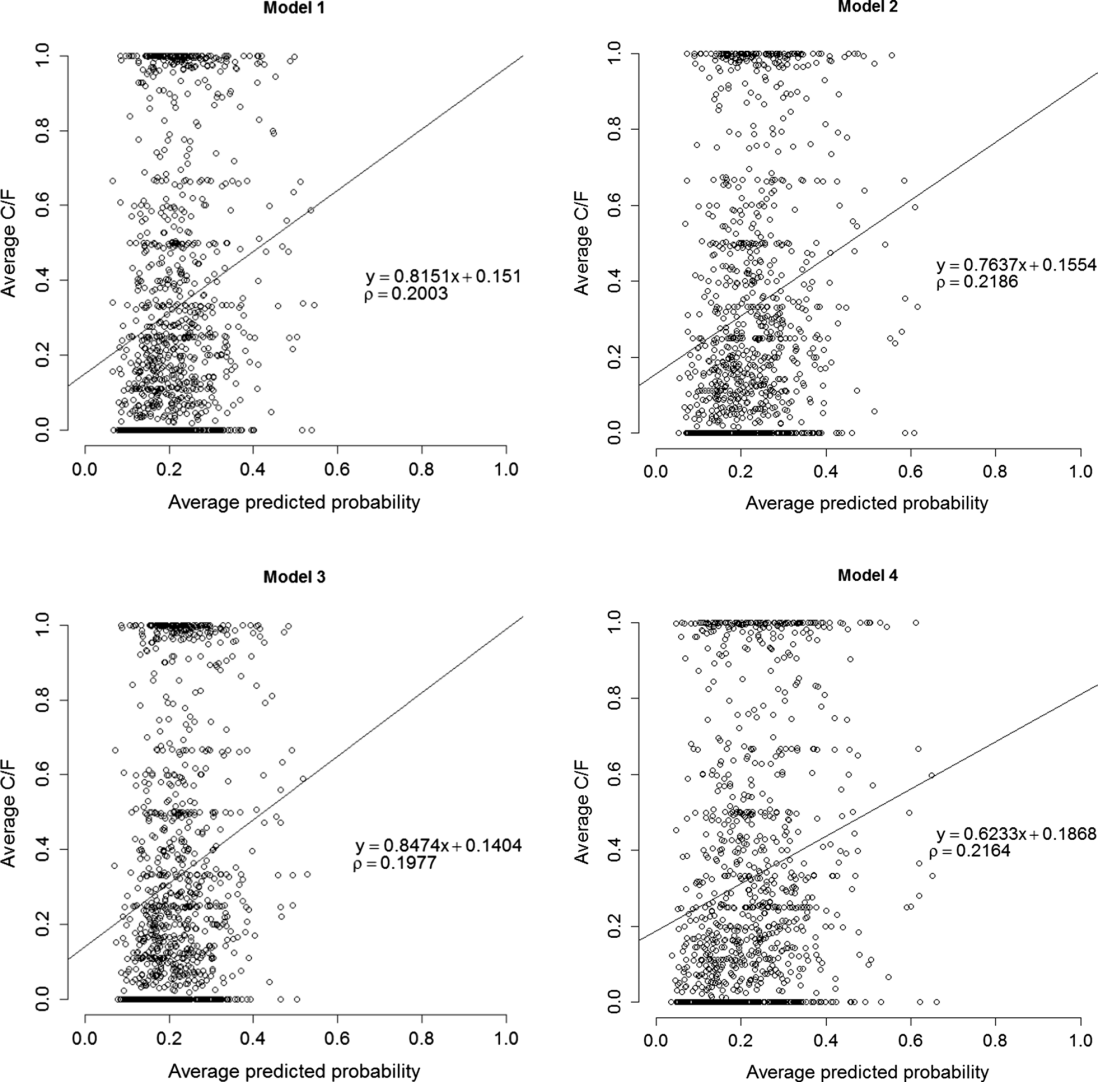
Weighted linear regression and correlation coefficients between the average observed number of cases over the number of susceptible feet (${C}/{F}$) and the average predicted probability for the observations. Regression coefficients smaller than 1 indicate over prediction

Figure 3 shows the weighted linear regression of the average observed number of cases over the number of susceptible feet ($C/F$) on the average predicted probability, and their correlation. Bias was smallest for models without the infectivity effect (Models 1 and 3), since regression coefficients of these models were closest to 1. The weighted correlations coefficients were higher for models with the infectivity effect (Models 2 and 4). Thus, models without an infectivity effect showed significantly lower bias, while models with an infectivity effect had a slightly higher accuracy. The higher bias for Model 4 compared to Model 2 agrees with results in Fig. 2, and confirms that omitting the random farm-by-period interaction inflates the variation in GEBV for infectivity.

### Basic reproduction ratio

The genomic estimated breeding values (GEBV) for susceptibility corrected for bias were approximately the same for Models 1, 2 and 4, and ranged from 0.49 to 2.81; the GEBV from Model 3 ranged from 0.50 to 2.53. A cow with a susceptibility GEBV of 0.50 is genetically about two times less susceptible than an average cow, whereas a cow with a susceptibility GEBV of 2.50 is genetically about 2.5 times more susceptible than an average cow. The GEBV for infectivity showed substantially more variation than the GEBV for susceptibility.

**Fig. 4:**
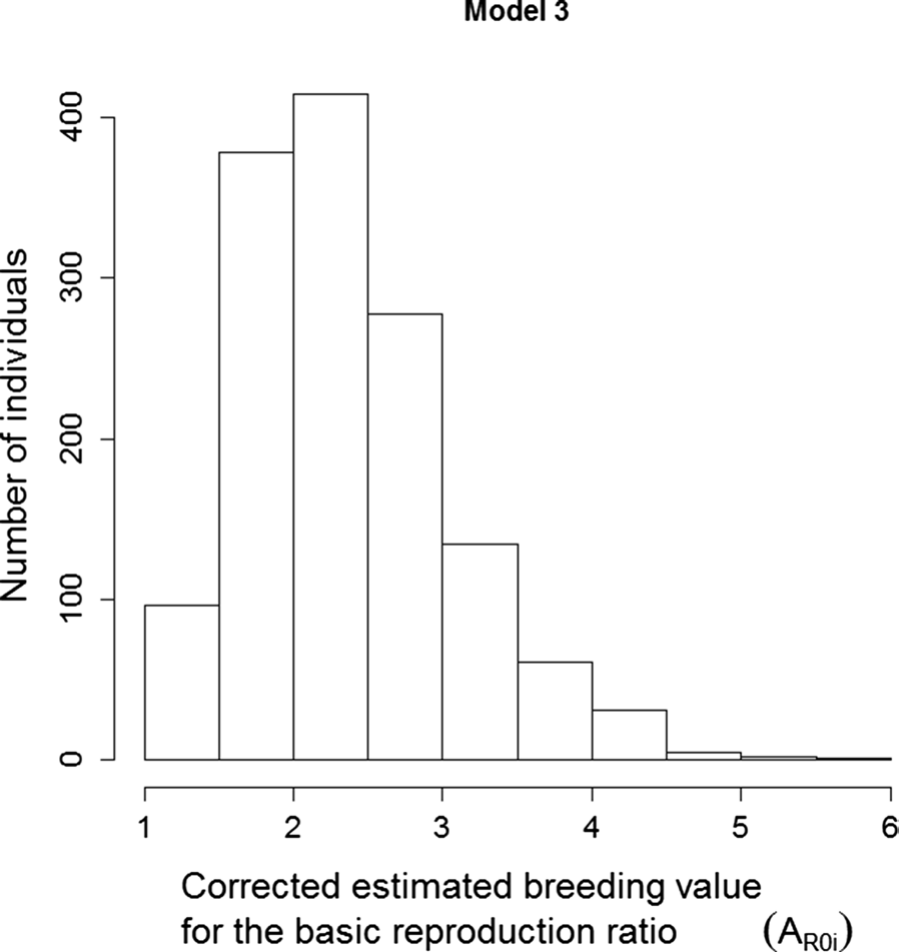
Histogram of the individual GEBV for the basic reproduction ratio corrected for bias for all genotyped cows, based on results from Model 3

We chose to illustrate the variation in GEBV for $R_{0}$ using results from Model 3, which is the most conservative model as judged by the estimated genetic variance in susceptibility (smallest value) and the bias. For this model, we calculated individual breeding values for the basic reproduction ratio ($\hat{A}_{{R_{0} ,i}}$) corrected for the bias that we found from the cross-validation. The average GEBV for susceptibility was 1.081, which is slightly higher than 1 because of the transformation from the log scale to the normal scale. In Model 3, there was no infectivity effect, thus infectivity was 1.00 for all individuals. With an average $R_{0}$ for DD of 2.36 on our farms [26], $c/\alpha$ was estimated at 2.18. GEBV for $R_{0}$ ranged from 1.089 to 5.515 (Fig. 4) (the GEBV not corrected for bias ranged from 0.62 to 6.68). This result indicates very substantial genetic variation in $R_{0}$. The expected prevalence in a population of individuals similar to the genetically best individual equals approximately $1 - \frac{1}{{R_{0} }} = 1 - \frac{1}{1.089} = 8.2\%$ [45], while the corresponding value for the genetically worst individual equals 81.9%.

The estimated additive genetic standard deviation for $R_{0}$ was 1.17 (this value is corrected for bias). This result shows that the current $R_{0}$ of 2.36 [26] is only about one genetic standard deviation greater than 1. Hence, this suggests that a genetic improvement of $R_{0}$ by a bit more than one genetic standard deviation would be sufficient to eradicate DD.

The approximate accuracy of $\hat{A}_{{R_{0} ,i}}$ follows from the ratio of the standard deviation of GEBV for $R_{0}$ over the additive genetic standard deviation of the true breeding values for $R_{0}$; $\rho_{{\hat{A}_{{R_{0} }} }} = \frac{{\sigma_{{\hat{A}_{{R_{0} }} }} }}{{\sigma_{{A_{{R_{0} }} }} }}$. This accuracy equals ~ 39%. To be conservative, we did not correct (i.e., shrink) the $\sigma_{{A_{{R_{0} }} }}$ in the denominator of this expression, while we did correct the numerator for the bias. Note that this accuracy is based on validation within a generation, rather than on validation forward in time. The accuracy based on validation forward in time is relevant for response to selection, and may be somewhat lower because genetic relationships may be a little weaker.

## Discussion

We estimated the additive genetic variation in host susceptibility, infectivity, and the basic reproduction ratio ($R_{0}$) for DD in dairy cattle. Furthermore, we calculated GEBV for susceptibility, infectivity, and $R_{0}$ for each animal. Four models were compared for their ability to predict whether a susceptible animal becomes infected. All four models included a genetic effect for susceptibility; Models 2 and 4 also included a genetic effect for infectivity, while Models 1 and 2 included an interaction term between farm and period. In all models, the estimates are corrected for the variation in exposure of the susceptible individuals to infected group mates via the offset of the model. The estimated additive genetic standard deviation for $R_{0}$ was large, ~ 1.17, and the mean $R_{0}$ (2.36) was only about one genetic standard deviation greater than the important threshold value of 1. Furthermore, GEBV for $R_{0}$ (corrected for bias) showed large variation, ranging from 1.089 to 5.515, and the approximate accuracy of GEBV for $R_{0}$ was ~ 0.39. These results show that genetic selection against DD is very promising; there is substantial heritable variation and a meaningful accuracy can be obtained from a limited amount of data.

Farm, parity, period, and months in milk of the focal cow were included in the models as fixed effects. The transmission rate parameter was 21% higher for parity 2 compared to parity 1, and 69% higher for parity greater than 2 compared to parity 1. The prevalence also increased with parity. This is in contrast with most previous studies where DD was most prevalent in first and second parity cows [15, 46]. For months in milk, the transmission rate parameter decreased by 4% per month in milk. This is in agreement with Argáez-Rodríguez et al. [46] who found that cows had the highest risk of getting DD in the first and third months of lactation, after which the risk decreased. The potential effect of parity and months in milk on the infectivity of a cow was not considered, because incorporating these factors in the summed effects of the infected group mates of a focal cow was methodologically too challenging.

We managed, only partly, to account for genetic effects on infectivity. For technical reasons, we included only the genetic effects of claws that were infected at the start of the observation interval ($t$) in the statistical model. However, the majority of the infection pressure originated from earlier infected claws that still contribute to transmission via the environment. We estimated a survival rate (λ) of the pathogen of 0.9 [26], meaning that 90% of the total infectious pressure originates from infectivity present in the environment from claws that were infected before the start of the observation interval. This suggests that we missed a large part of the potential heritable variation in infectivity. Hence, the relevance of genetic variation in infectivity for DD may be larger than suggested by the estimates presented here (Table 3). Unfortunately, in the statistical software we did not manage to keep track of all the weighted genotypes (i.e., weighing the genotypes of the infected individuals for their contribution to the environment). Nevertheless, the accuracy of predicting the phenotype from the cross-validation for the model with genetic effects of infectivity (Model 2) was 9% higher than the accuracy for the corresponding model without these effects (Model 1). Thus, even including a small proportion of the apparent variation in infectivity in the model appears to increase the accuracy of GEBV.

Estimates of infectivity show relatively larger standard errors because variation in infectivity must be estimated indirectly, unlike variation in susceptibility. Infectivity estimates are based on the number of susceptible group mates of an infectious individual that become infected, and on differences in genotype among the infected group mates at different time points. When there are multiple infected group mates, the accuracy of the infectivity estimates decreases [7]. Especially in large groups, as in this study (~ 100 cows), more records and groups are needed to estimate genetic variation in infectivity accurately [47]. This issue is very similar to the estimation of indirect genetic effects from large groups. In addition, we observed DD transmission for a relatively short period of time. More accurate estimation of genetic variation in infectivity requires data that are recorded over longer time periods (i.e., to be able to observe the entire infectious period for the majority of the cows), more groups (herds), a better statistical model (i.e., inclusion of the genetic effects from earlier infected cows of which the pathogens are still present in the environment), and the inclusion of infectivity effects of cows without genotypes. The latter could be done using single-step GBLUP [48].

In the first two models, we included an interaction between farm and period to account for non-genetic effects of infectivity. This interaction serves to avoid overestimation of the genetic variance in infectivity [49], similar to the inclusion of a random group effect in the analysis of indirect genetic effects [38, 50]. The genetic effect for infectivity and the interaction term were partly confounded, because both terms have an effect on the number of susceptible animals that become a case within a certain period on a certain farm. However, confounding is not complete because of genetic relationships between the infected animals across farms and periods. Our results also suggest that inclusion of a random farm-by-period effect is essential to avoid overestimation of the genetic variation due to infectivity.

The estimated variances for susceptibility (genetic and non-genetic) were lower for Model 3 that included neither a genetic effect for infectivity nor an interaction between farm and period. Anacleto et al. [7] showed that estimates for susceptibility are less accurate when genetic variation in infectivity is not accounted for. Indeed, we also found a slightly higher correlation in the cross-validation when infectivity was included in the model, although this was accompanied by an inflation of the GEBV, as shown by the regression coefficients in Fig. 3. However, inflation of GEBV can be remedied by shrinking them based on the results of cross-validation, whereas a reduction in correlation cannot. Therefore, even when genetic variation in infectivity is small, it might be beneficial to include infectivity in the model to more accurately estimate susceptibility GEBV [7].

In the cross-validation, we estimated a weighted correlation of about 0.2 between the observed and predicted number of cases over the number of susceptible feet. This value can be used to approximate the accuracy of the GEBV ($r_{{g,\widehat{g}}}$) [51],

8 $$ r_{{g,\widehat{g}}} \approx \frac{{r_{{p,\widehat{g}}} }}{{\sqrt {h^{2} } }}, $$

where, $r_{{p,\widehat{g}}}$ is the correlation between the observations and the predictions, and $h^{2}$ is the heritability of the trait. Heritability estimates for digital dermatitis from previous studies range from 0.05 to 0.29, depending on the model used [52]. Assuming a heritability of 0.28 for DD [53], the accuracy of the predicted number of cases is 0.37.

In general, studies on genetic variability of infectious diseases commonly focus on individual differences in susceptibility only, and those differences are estimated with linear models that ignore variation in exposure between individuals. In this study, we used a GLMM founded on epidemiological theory to estimate genetic variability in susceptibility and infectivity. An important advantage of models founded on epidemiological theory is that they provide estimates for epidemiological parameters, such as $R_{0}$, that are interpretable in the context of infectious disease dynamics. Our results, for example, show that $R_{0}$ is only slightly more than one genetic standard deviation away from the threshold value of 1, which suggests that eradication of DD by genetic improvement is *in principle* feasible. Further work is needed to quantify the benefits of GLMM based on epidemiological theory over simpler linear models, to better account for the potential genetic variation in infectivity via the environment, and to include genetic variation in the duration of the infectious period.

## Conclusions

Genetic variance components for susceptibility and infectivity for digital dermatitis were estimated with four generalized linear mixed models. We managed, only partly, to account for genetic effects on infectivity. We estimated GEBV for the basic reproduction ratio from a (conservative) model including genetic effects for susceptibility only. GEBV for $R_{0}$ were corrected for bias, and showed substantial variation, ranging from 1.089 to 5.515. The mean $R_{0}$ (2.36) was only about one genetic standard deviation greater than 1. Based on cross-validation between farms, the approximate accuracy of GEBV for $R_{0}$ was 0.39, in spite of the relatively small dataset of only 12 genotyped herds. These results suggest that lowering prevalence of DD by selective breeding is very promising.

## Supplementary information

**Additional file 1.** Fixed effect estimates. Table of fixed effect estimates and standard errors on the log scale from Model 1.

## Data Availability

Datasets used and analysed during the current study are available from the corresponding author on reasonable request.

## Appendix

### Cross-validation

In the 12-fold cross-validation, we censored the number of cases in each period and predicted for each susceptible animal the number of cases over the number of susceptible feet ($C_{i} /F_{i}$) based on information of the 11 other farms. In general, the predicted probability for the number of cases over the number of susceptible feet can be calculated with the estimated effects. Because of the complementary log–log link function, these effects need to be back-calculated to the original scale:

9 $$ \widehat{P}_{i} \left( t \right) = 1 - e^{{ - e^{{\sum \widehat{FE} + \widehat{{{ \log }\left( {\gamma_{i} } \right)}} + { \log }\left( {\frac{{I_{tot} \left( t \right) + E\left( t \right)}}{N\left( t \right)}\Delta t} \right)}} }} , $$

when the genetic effect for infectivity was not included (Models 1 and 3), and

10 $$ \widehat{{P_{i} }}\left( t \right) = 1 - e^{{ - e^{{\sum \widehat{FE} + {\text{l}}\widehat{{{\text{og}}\left( {\gamma_{i} } \right)}} + \frac{1}{{I_{g} \left( t \right)}}\mathop \sum \limits_{j} {\text{l}}\widehat{{{\text{og}}\left( {\varphi_{j} } \right)}} + { \log }\left( {\frac{{I_{tot} \left( t \right) + E\left( t \right)}}{N\left( t \right)}\Delta t} \right)}} }} , $$

when the genetic effect for infectivity was included (Models 2 and 4).

In Eqs. (9) and (10), $\widehat{{P_{i} }}\left( t \right)$ is the predicted probability for the number of cases over the number of susceptible feet for animal $i$ in the time from $t$ to $t + 1$, $\sum FE$ is the sum of the estimates for the fixed effects, i.e., the estimates for the intercept, farm $k$, period $\tau$, parity $l$, and months in milk. The $\widehat{{{ \log }\left( {\gamma_{i} } \right)}}$ is the estimated genetic effect for susceptibility on the log scale and $\mathop \sum \nolimits_{j} \widehat{{{ \log }\left( {\varphi_{j} } \right)}}$ is the sum of the estimated genetic effects for infectivity of the infected group mates of the susceptible individual on the log scale. The last term in Eqs. (9) and (10) is the offset. In the 12-fold cross-validation, the cases ($C$) of one entire farm were censored from the dataset, therefore, the random effects for the interaction between farm and period, and non-genetic animal effect for animal $i$ could not be estimated for this farm. Thus, these random effects did not contribute to the predicted probabilities, and they are not included in Eqs. (9) and (10).

To validate the estimated genetic effects, we wanted to estimate the probability that an animal would be a case during an interval independent of the fixed effects in the model. We achieved this independence by standardizing both $\widehat{{P_{i} }}\left( t \right)$ and $C_{i} \left( t \right)/F_{i} \left( t \right)$. We obtained the regression coefficients for the fixed effects for each of the four models that were applied to the full dataset where no data was censored. With these regression coefficients, we calculated the average value of summed fixed effects $\left( {\overline{\sum FE} } \right)$. The $\overline{\sum FE}$ can be interpreted as a standard/average farm and a standard/average cow. This $\overline{\sum FE}$ was used in Eqs. (9) and (10) instead of the estimated fixed effects:

11 $$ \widehat{{P_{i} }}\left( t \right)^{*} = 1 - e^{{ - e^{{\overline{\sum FE} + {\text{l}}\widehat{{{\text{og}}\left( {\gamma_{i} } \right)}} + { \log }\left( {\frac{{I_{tot} \left( t \right) + E\left( t \right)}}{N\left( t \right)}\Delta t} \right)}} }} , $$

for Models 1 and 3, and

12 $$ \widehat{{P_{i} }}\left( t \right)^{*} = 1 - e^{{ - e^{{\overline{\sum FE} + {\text{l}}\widehat{{{\text{og}}\left( {\gamma_{i} } \right)}} + \frac{1}{{I_{g} \left( t \right)}}\mathop \sum \limits_{j} \widehat{{{ \log }\left( {\varphi_{j} } \right)}} + { \log }\left( {\frac{{I_{tot} \left( t \right) + E\left( t \right)}}{N\left( t \right)}\Delta t} \right)}} }} , $$

for Models 2 and 4.

Here, $\widehat{{P_{i} }}\left( t \right)^{*}$ is the predicted probability for the number of cases over the number of susceptible feet for animal $i$ in a period, as if it were an average cow with an average parity and months in milk, during a standard period, on a standard farm. Note that the genetic susceptibility and infectivity did differ between cows, and thus had an effect on the predictions.

Similarly, we wanted to standardize the observed cases over the number of susceptible claws $\left( {C_{i} \left( t \right)/F_{i} \left( t \right)} \right)$, so that they would be independent of the fixed effects that contributed to that observation. The observations were transformed to the complementary log–log scale so that they were linear in the effects:

13 $$ \begin{aligned} & { \log }( - \log \left( {1 - {\raise0.7ex\hbox{${C_{i} \left( t \right)}$} \!\mathord{\left/ {\vphantom {{C_{i} \left( t \right)} {F_{i} \left( t \right)}}}\right.\kern-0pt} \!\lower0.7ex\hbox{${F_{i} \left( t \right)}$}}} \right) \\ & \quad= \sum FE + \log \left( {\gamma_{i} } \right) + {\text{log}}\left( {\frac{{I_{tot} \left( t \right) + E\left( t \right)}}{N\left( t \right)}\Delta t} \right) \\ \end{aligned} $$

for Models 1 and 3, and,

14 $$  \begin{aligned} & { \log }( - \log \left( {1 - {\raise0.7ex\hbox{${C_{i} \left( t \right)}$} \!\mathord{\left/ {\vphantom {{C_{i} \left( t \right)} {F_{i} \left( t \right)}}}\right.\kern-0pt} \!\lower0.7ex\hbox{${F_{i} \left( t \right)}$}}} \right) \\ & \quad= \sum FE + \log \left( {\gamma_{i} } \right) + \frac{1}{{I_{g} \left( t \right)}}\mathop \sum \limits_{j} \log \left( {\varphi_{j} } \right) \\&\qquad+ { \log }\left( {\frac{{I_{tot} \left( t \right) + E\left( t \right)}}{N\left( t \right)}\Delta t} \right) \\ \end{aligned}$$

for Models 2 and 4.

Next, the summed fixed effects $\sum FE$ in Eqs. (13) and (14) were replaced with the average value of the summed fixed effects $\left( {\overline{FE} } \right)$, and back-calculated to the original scale to obtain the observed number of cases over the number of susceptible feet independent of the fixed effects $\left( {(C_{i} \left( t \right)/F_{i} \left( t \right))^{*} } \right)$, for all models:

15 $$ \left( {C_{i} \left( t \right)/F_{i} \left( t \right)} \right)^{*} = 1 - e^{{ - e^{{\left( {\left( {{ \log }\left( { - log\left( {1 - {\raise0.7ex\hbox{${C_{i} \left( t \right)}$} \!\mathord{\left/ {\vphantom {{C_{i} \left( t \right)} {F_{i} \left( t \right)}}}\right.\kern-0pt} \!\lower0.7ex\hbox{${F_{i} \left( t \right)}$}}} \right)} \right)} \right) - \sum FE + \overline{\sum FE} } \right)}} }} . $$

Here, $(C_{i} \left( t \right)/F_{i} \left( t \right))^{*}$ is the observed number of cases over the number of susceptible feet for animal $i$ in a period, as if observed on an average cow with an average parity and months in milk, on a standard farm. Again, the genetic susceptibility and infectivity did differ between cows (see Eqs. (13) and (14)), and affected the dependent variable.

We calculated weighted correlation coefficients between the “corrected” observed number of cases over the number of susceptible feet ($\overline{{\left( {C_{i} \left( t \right)/F_{i} \left( t \right)} \right)^{*} }}$) and the “corrected” predicted probabilities ($\overline{{\widehat{{P_{i} }}\left( t \right)^{*} }}$), averaged over the number of times an animal was susceptible at the start of an interval, and this number was used as the weight.
