# Supplementary material for: Genetic parameters and genomic breeding values for digital dermatitis in Holstein Friesian dairy cattle: host susceptibility, infectivity and the basic reproduction ratio
Source: Genet Sel Evol. 2019 Nov 20;51:67. doi: 10.1186/s12711-019-0505-3 (PMC6865030; doi:10.1186/s12711-019-0505-3)
Supplement: Supplementary file 1 — Additional file 1. Fixed effect estimates. Table of fixed effect estimates and standard errors on the log scale from Model 1. [file 12711_2019_505_MOESM1_ESM.docx]

**Additional file 1.**

**Fixed effect estimates**

| **Model term** | **Level** | **Effect^1^** | **Standard error^1^** |
| --- | --- | --- | --- |
| Mu |  | -1.8480 | 0.2777 |
| Farm | A | 0.3562 | 0.3241 |
|  | B | 0.4260 | 0.3282 |
|  | C | -0.1308 | 0.3101 |
|  | D | -0.1511 | 0.3257 |
|  | E | -0.1477 | 0.3380 |
|  | F | 0.4791 | 0.3275 |
|  | G | -0.1176 | 0.3397 |
|  | H | 0.0000 | 0.0000 |
|  | I | 0.3636 | 0.3552 |
|  | J | 0.5933 | 0.3574 |
|  | K | -0.0115 | 0.3337 |
|  | L | 0.2493 | 0.3083 |
| Period | 1 | -0.6831 | 0.2350 |
|  | 2 | -0.3417 | 0.2348 |
|  | 3 | -0.7532 | 0.2410 |
|  | 4 | -0.1702 | 0.2326 |
|  | 5 | 0.0000 | 0.0000 |
|  | 6 | -0.5456 | 0.2484 |
|  | 7 | -0.6752 | 0.2396 |
|  | 8 | -0.6096 | 0.2385 |
|  | 9 | -0.3894 | 0.2415 |
|  | 10 | -0.5207 | 0.2459 |
| Parity | 1 | -0.7614 | 0.1156 |
|  | 2 | -0.5138 | 0.1254 |
|  | >2 | 0.0000 | 0.0000 |
| Months in milk |  | -0.0667 | 0.0106 |
| Farm.Period | A.1 | -0.2227 | 0.3076 |
|  | A.2 | -0.3074 | 0.3138 |
|  | A.3 | -0.8054 | 0.3483 |
|  | A.4 | -0.1761 | 0.2870 |
|  | A.5 | 0.3772 | 0.2934 |
|  | A.6 | -0.1091 | 0.3497 |
|  | A.7 | 0.6294 | 0.2966 |
|  | A.8 | 0.1793 | 0.3416 |
|  | A.9 | 0.3448 | 0.3036 |
|  | A.10 | 0.0090 | 0.3526 |
|  | B.1 | 0.5796 | 0.2774 |
|  | B.2 | -0.4217 | 0.3131 |
|  | B.3 | -0.6965 | 0.3218 |
|  | B.4 | -0.5023 | 0.2909 |
|  | B.5 | 0.2689 | 0.2760 |
|  | B.6 | 0.1208 | 0.3325 |
|  | B.7 | -0.0252 | 0.3140 |
|  | B.8 | 0.1371 | 0.3007 |
|  | B.9 | 0.4781 | 0.2881 |
|  | B.10 | 0.0611 | 0.3280 |
|  | C.1 | -0.4447 | 0.3268 |
|  | C.2 | 0.4165 | 0.2790 |
|  | C.3 | -0.2360 | 0.3314 |
|  | C.4 | 0.0561 | 0.2908 |
|  | C.5 | -0.3874 | 0.2985 |
|  | C.6 | 0.1356 | 0.3129 |
|  | C.7 | 0.0251 | 0.3161 |
|  | C.8 | 0.2090 | 0.3002 |
|  | C.9 | -0.1109 | 0.3043 |
|  | C.10 | 0.3367 | 0.2948 |
|  | D.1 | -0.5709 | 0.3227 |
|  | D.2 | 0.4564 | 0.2870 |
|  | D.3 | 0.6555 | 0.3203 |
|  | D.4 | 0.6642 | 0.2850 |
|  | D.5 | -0.1846 | 0.3165 |
|  | D.6 | 0.2624 | 0.3176 |
|  | D.7 | 0.0317 | 0.3389 |
|  | D.8 | -1.0320 | 0.3722 |
|  | D.9 | -0.1407 | 0.3026 |
|  | D.10 | -0.1419 | 0.3231 |
|  | E.1 | 0.1963 | 0.3275 |
|  | E.2 | -0.2730 | 0.3431 |
|  | E.3 | -0.2004 | 0.3531 |
|  | E.4 | -0.6142 | 0.3410 |
|  | E.5 | 0.2053 | 0.3031 |
|  | E.6 | 0.3839 | 0.3485 |
|  | E.7 | 0.4751 | 0.3465 |
|  | E.8 | 0.1923 | 0.3493 |
|  | E.9 | 0.1332 | 0.3313 |
|  | E.10 | -0.4984 | 0.3732 |
|  | F.1 | 0.0679 | 0.2932 |
|  | F.2 | -0.4763 | 0.3125 |
|  | F.3 | -0.3318 | 0.3091 |
|  | F.4 | -0.6448 | 0.3037 |
|  | F.5 | -0.5699 | 0.2817 |
|  | F.6 | 0.0000 | 0.5117 |
|  | F.7 | 0.6032 | 0.3063 |
|  | F.8 | 0.4640 | 0.3010 |
|  | F.9 | 0.2428 | 0.2992 |
|  | F.10 | 0.6449 | 0.3015 |
|  | G.1 | -0.0731 | 0.3163 |
|  | G.2 | 0.7544 | 0.2983 |
|  | G.3 | -0.4270 | 0.4106 |
|  | G.4 | 0.4901 | 0.3056 |
|  | G.5 | 0.2347 | 0.3393 |
|  | G.6 | -0.4255 | 0.3915 |
|  | G.7 | -0.4203 | 0.3613 |
|  | G.8 | 0.1078 | 0.3251 |
|  | G.9 | -0.1754 | 0.3373 |
|  | G.10 | -0.0657 | 0.3404 |
|  | H.1 | -0.2974 | 0.3007 |
|  | H.2 | 0.5587 | 0.2628 |
|  | H.3 | 0.3061 | 0.2983 |
|  | H.4 | -0.2564 | 0.2727 |
|  | H.5 | 0.0252 | 0.2673 |
|  | H.6 | 0.1994 | 0.2907 |
|  | H.7 | -0.2270 | 0.3046 |
|  | H.8 | 0.3766 | 0.2742 |
|  | H.9 | -0.4962 | 0.2987 |
|  | H.10 | -0.1890 | 0.2820 |
|  | I.1 | -0.0149 | 0.3098 |
|  | I.2 | 0.3166 | 0.2904 |
|  | I.3 | 0.1083 | 0.3362 |
|  | I.4 | 0.2155 | 0.2857 |
|  | I.5 | -0.3752 | 0.3203 |
|  | I.6 | -0.1135 | 0.3173 |
|  | I.7 | -0.0461 | 0.3253 |
|  | I.8 | 0.3815 | 0.3021 |
|  | I.9 | -0.1874 | 0.3275 |
|  | I.10 | -0.2848 | 0.3311 |
|  | J.1 | 0.1092 | 0.2970 |
|  | J.2 | -0.6813 | 0.3315 |
|  | J.3 | 0.1819 | 0.2962 |
|  | J.4 | 0.8898 | 0.2839 |
|  | J.5 | 0.2946 | 0.3390 |
|  | J.6 | 0.2315 | 0.3204 |
|  | J.7 | -0.4749 | 0.3600 |
|  | J.8 | 0.1496 | 0.3005 |
|  | J.9 | -0.4413 | 0.3323 |
|  | J.10 | -0.2591 | 0.3375 |
|  | K.1 | 0.0224 | 0.2991 |
|  | K.2 | 0.0519 | 0.2977 |
|  | K.3 | 0.5125 | 0.3088 |
|  | K.4 | 0.5625 | 0.2877 |
|  | K.5 | 0.1144 | 0.3205 |
|  | K.6 | -0.5235 | 0.3424 |
|  | K.7 | 0.0150 | 0.3071 |
|  | K.8 | -0.7551 | 0.3517 |
|  | K.9 | 0.0000 | 0.5117 |
|  | K.10 | 0.0000 | 0.5117 |
|  | L.1 | 0.6482 | 0.2696 |
|  | L.2 | -0.3948 | 0.3110 |
|  | L.3 | 0.9330 | 0.2757 |
|  | L.4 | -0.6844 | 0.3112 |
|  | L.5 | -0.0033 | 0.2709 |
|  | L.6 | -0.1619 | 0.3276 |
|  | L.7 | -0.5860 | 0.3038 |
|  | L.8 | -0.4100 | 0.2881 |
|  | L.9 | 0.3531 | 0.2682 |
|  | L.10 | 0.3062 | 0.2957 |

^a^Estimates from Model 1, estimates and standard errors are on the log scale.
